# Nutritional and Therapeutic Strategies in Paediatric Phenylketonuria: A Narrative Literature Review

**DOI:** 10.3390/nu18091347

**Published:** 2026-04-24

**Authors:** Holly Jones, Eugen-Matthias Strehle

**Affiliations:** 1 School of Medicine, University of Sunderland, Sunderland SR1 3SD, UK; 2 The Medical School, Newcastle University, Newcastle upon Tyne NE2 4HH, UK

**Keywords:** phenylketonuria, sapropterin dihydrochloride, phenylalanine hydroxylase, diet, pegvaliase, adherence

## Abstract

Phenylketonuria (PKU) is an autosomal recessive disorder characterised by an inborn error of phenylalanine (Phe) metabolism. Such errors are attributed to pathogenic gene variants causing phenylalanine hydroxylase (PAH) deficiency, impairing the hydroxylation of phenylalanine to tyrosine in the Phe metabolic pathway. This defect leads to plasma Phe concentrations above the normal range. If untreated, hyperphenylalaninemia can adversely affect brain function, leading to severe intellectual disability and seizures. Since 1969, the newborn dried blood spot test has remained the main method of early screening and diagnosis for PKU. The primary therapeutic management is a lifelong phenylalanine-restricted diet with the aim of decreasing plasma Phe levels. The recommended diet consists of avoiding high-protein foods such as meat, fish, eggs and nuts, and can be supplemented with high-protein medical formulas which are low in phenylalanine. Pharmacological interventions such as sapropterin, sepiapterin and pegvaliase can also be used as treatment adjuncts in patients with PKU. Currently, small-molecule inhibitors reducing renal phenylalanine reabsorption are being explored as a potential therapeutic intervention. Furthermore, novel gene-editing techniques are under evaluation as potential curative strategies, with preclinical studies showing promising results in correcting pathogenic phenylalanine hydroxylase variants. This non-systematic review synthesises current literature on the management of PKU, with a focus on dietary interventions and recommendations.

## 1. Background

Phenylketonuria (PKU) is an autosomal recessive disorder characterised by an inborn error of phenylalanine metabolism. PKU was first described by Asbjørn Følling in 1934 in two siblings with severe intellectual disability [1]. The siblings were reported to have an unusual “musty” odour in their urine, which Følling later found to contain phenylpyruvic acid. This abnormal finding reflected a defect in phenylalanine metabolism, a term that was eventually coined as phenylketonuria.

**Figure 1:**
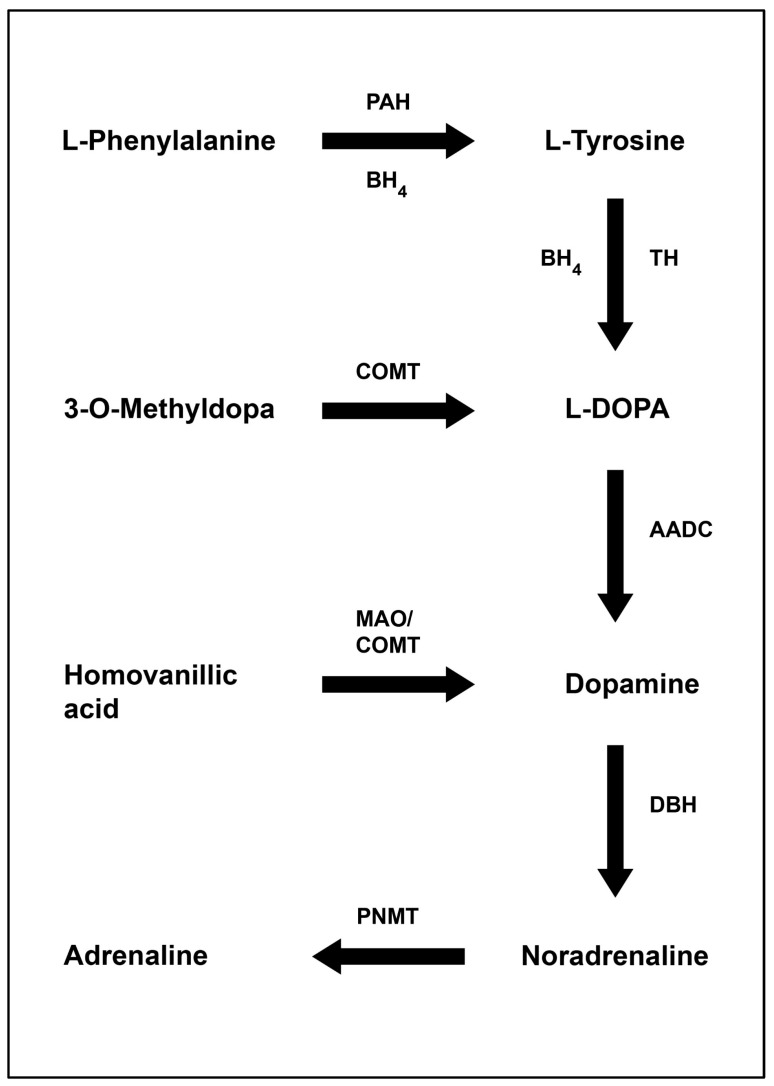
Diagram of phenylalanine metabolism demonstrating its importance for neurotransmitter synthesis (AADC = aromatic L-amino acid decarboxylase, BH4 = tetrahydrobiopterin, COMT = catechol-O-methyltransferase, DBH = dopamine beta-hydroxylase, PAH = phenylalanine hydroxylase, PNMT = phenylethanolamine-*N*-methyltransferase, and TH = tyrosine hydroxylase).

When metabolised, phenylalanine (Phe) is hydroxylated to form tyrosine by the enzyme phenylalanine hydroxylase (PAH) [2]. In PKU, mutations in the PAH gene render the enzyme ineffective and thus lead to hyperphenylalaninemia (HPA) [2]. As shown in Figure 1, the Phe metabolic pathway requires the presence of the cofactor tetrahydrobiopterin BH4. To facilitate this hydroxylation reaction, BH4 is oxidised to quinonoid dihydrobiopterin (qBH2), undergoing continuous recycling back to BH4 via the dihydropterin reductase enzyme. Tyrosine can then be metabolised to melanin, dopamine, noradrenaline and adrenaline.

Historically, PKU has been classified into classical, moderate and mild phenotypes, which were defined by pre-treatment levels of Phe [3]. Such levels are >1200 μmol/L in classical PKU, 600–1200 μmol/L in moderate PKU and <600 μmol/L in mild PKU [3]. In the recent first revision of European PKU Guidelines [4], an amended classification has been suggested, which considers the availability of new treatments such as co-factor BH4 therapy. Individuals with PAH deficiency may now be recognised as (a) not requiring treatment (Phe < 360 μmol/L); (b) requiring treatment, co-factor responsive, or (c) requiring treatment, co-factor unresponsive [4].

In 1961, Robert Guthrie developed a test that could detect elevated Phe levels in the blood [5]. This has now been adopted globally as the primary screening method for PKU, termed the newborn blood spot screening programme. It is partially due to this early detection programme that those with PKU now experience improved neurocognitive and educational outcomes [6]. Management for PKU was first proposed in 1953 by Horst Bickel, who treated a 4-year-old girl with PKU by restricting dietary Phe intake [7]. Bickel observed a subsequent reduction in plasma Phe and disappearance of the characteristic “musty” urinary odour. Dietary Phe restriction still remains the cornerstone of management in PKU and aims to effectively reduce Phe levels to within the target range [6,8].

Phe target ranges are classified by European PKU guidelines as within 120–360 µmol/L for children up to the age of 12, and 120–600 µmol/L for children above this age [6,9]. Maintaining adequate metabolic control is crucial due to the potential impact of raised Phe concentrations. Hyperphenylalaninemia has been shown to worsen neurological outcomes [9,10,11], a factor which has been reflected in the literature as lower IQ [11] and poorer memory and processing skills [12]. This risk increases in children and adolescents due to the sensitivity of the developing brain to permanent injury [9]. To mitigate such risks, dietary management is recommended to be lifelong and begin as early as possible [13,14,15]. The National PKU Collaborative Study during 1967–1983 provided evidence in support of this recommendation. The study followed 211 newborn infants to evaluate the importance of early and consistent treatment of PKU [13] by providing longitudinal follow-up. Early initiation of treatment and lower Phe levels during childhood were correlated with better cognitive outcomes and academic performance at age 12 [14], highlighting the importance of prompt recognition and treatment of PKU in children in long-term outcomes [13,16,17].

Dietary management is characterised by the consumption of low-Phe foods with supplementation of medical formulas acting as protein substitutes [6]. Medical formulas include Phe-free L-amino acid supplements and glycomacropeptide (GMP) based substitutes [6,18] and are imperative in maintaining adequate growth and preventing protein deficiency [6]. The use of supplementary large neutral amino acids (LNAAs), which inhibit Phe absorption in the gut [19] have also been trialled alongside dietary management. Though these have shown evidence of reducing Phe levels [19], they are not recommended for children below 12 years of age [16].

Whilst lifelong dietary measures remain the mainstay of management in PKU, pharmacological therapies such as sapropterin dihydrochloride, a synthetic form of BH4 enzyme, have been developed to improve metabolic control in BH4-responsive individuals [20]. Sapropterin has been shown to decrease blood Phe levels in paediatric populations [21] and has now received approval for treatment in those under 4 years old [22]. More recently, Pegvaliase, an enzyme substitution therapy, has altered the treatment landscape in PKU and offers the potential to reduce dietary Phe restriction in a proportion of patients.

This narrative literature review aims to provide an overview of the management of PKU in the paediatric population, such as dietary Phe restriction, protein substitutes/medical formulas and natural protein intake. Furthermore, the review will aim to discuss the barriers to dietary adherence and explore emerging therapies.

## 2. Breastfeeding and Management in Infancy

As recommended by European PKU guidelines, dietary management of PKU should begin by the age of 10 days in infants with blood Phe levels above 360 μmol/L, as early treatment is associated with improved neurological outcomes [6,14]. Although the literature is generally consistent regarding treatment initiation in infants with blood Phe levels > 600 μmol/L, evidence surrounding treatment at Phe levels between 360 and 600 μmol/L is variable [23,24]. Thus, treatment is recommended within this range during the first 12 years of life. Continuation of treatment is dependent on blood Phe levels beyond this age. If blood Phe levels remain below 600 μmol/L, it is possible that these patients may no longer require treatment [4].

Phe requirements during infancy are fulfilled with breast milk or standard infant formula [25] supplemented by Phe-free formula [6,26]. Breastfeeding is well-evidenced in PKU [27] and is a preferable source of Phe due to factors such as increased mother–infant bonding, greater convenience and content of polyunsaturated fatty acids [6,28]. Protein content in human breast milk is variable, ranging from around 14–16 g/L at birth to 7–8 g/L after 6 months [29]. This variability means that monitoring of Phe levels in infants who are breastfeeding is of particular importance [30]. Thus, though breastfeeding is beneficial, intake in infants with PKU is reduced in comparison to normal infants due to the risk of adverse impact on metabolic control [31].

A variety of methods can be used in infant feeding, with the most frequently used method by European centres being to breastfeed after a set amount of Phe-free formula has been given to the infant [27]. Another approach is interchanging feeds with both Phe-free formula and breast milk. Both methods have been shown to be nutritionally adequate, though there are few studies comparing the methods and their longitudinal impact on metabolic control and growth outcomes [27]. As feeding an infant with PKU can be challenging, support for mothers during breastfeeding and weaning is recommended [28].

Weaning usually occurs between 17 and 26 weeks [27,28], with this time frame being associated with better acceptance and less food refusal compared to early or delayed weaning [32]. Though weaning in PKU can present with challenges [31,32], the process should largely reflect that of healthy controls. Weaning foods are commonly low-protein foods containing Phe of ≤75 mg/100 g, and can be mixed with milk, cream or butter to increase their energy content [28]. As in normal weaning practices, the food is given as a semi-solid puree and often comprises low-Phe fruits and vegetables [28]. Over time, measured natural protein foods such as baby cereal, yoghurt, and baby rice can be introduced, followed by finger foods such as soft fruits and low-protein toast as part of the exchange system [28]. In the process of reducing Phe-free formula during weaning, there is a reduction in energy intake [31]. Thus, it is recommended that a more concentrated second-stage protein substitute be introduced from 6 months of age [28] to maintain protein intake.

## 3. Dietary Phenylalanine Restriction

Lifelong dietary management in PKU aims to prevent the build-up of plasma Phe whilst ensuring adequate nutrient intake [28] for growth and tissue repair [6]. Phe is an essential amino acid forming protein [33]; thus, high-protein food items are restricted to maintain Phe levels within the normal range [6,28]. Restricted food products include meat, nuts, soya, aspartame and cheese [6,28].

In PKU, consumption of natural protein is decreased to approximately 25% or less compared to the general population [28], with the majority of dietary protein coming from Phe-free L-amino acid supplements [34]. Total protein intake should ideally exceed the recommendations given for the general population [6] and should be based on ideal body weight rather than actual body weight [28,34,35].

Phe tolerance generally decreases with age in those with PKU [6,35], from 55 mg/kg/day in infancy up to 3 months [6] to 14 mg/kg/day at 10 years of age [34,36]. Establishing tolerance ranges ensures the avoidance of unnecessary restriction of natural proteins [34,36] and consequently reduces the risk of diminished growth and osteopenia [37]. Thus, a higher Phe tolerance may not only increase variability within the diet but also provide nutritional advantages [28]. However, it should be noted that tolerance is individual and dependent on factors such as PKU severity, dietary adherence, growth and catabolic state [28].

There are multiple systems used to calculate allocated Phe intake, such as the Phe exchange system, where foods are counted as “exchanges” and represent a fixed amount of Phe, e.g., 50, 25 or 20 mg [28]. Alternatively, 1 g of natural protein, representing one exchange, is calculated as 50 mg of Phe [28,35]. In these methods, there is also the inclusion of “exchange-free” foods, referring to foods that are permitted without restriction [16]. These include most foods containing ≤0.5 mg/100 g [28,38] and fruit and vegetables containing less than 75 mg/100 g [28,38]. A study by Weetch. et al. provides a comprehensive analysis of the phenylalanine content of fruit, vegetables and other miscellaneous food items [39]. Foods containing <50 mg/100 g of Phe include fruit such as apples, mangoes, guava and oranges, and vegetables such as aubergines, onions, carrots and celery. In contrast, foods containing higher levels of Phe (>75 mg/100 g) include fruits such as dried figs and passionfruit, and vegetables including garden peas, spinach and spring greens. The literature has demonstrated that consumption of exchange-free foods does not negatively impact blood Phe control [40,41,42] and can also provide beneficial fibre [40].

Another approach is the precise calculation of Phe content in foods with the aid of paper lists or apps [28]. Usage of systems differs depending on geographical location. For example, a 50 mg Phe exchange system is implemented in the UK, with the United States and Australia using a 15 mg Phe exchange system [43]. Availability of studies comparing the outcomes of different dietary systems in PKU remains scarce, and the lack of standardisation in prescribing practices may make it difficult to directly compare growth outcomes across paediatric populations.

European PKU guidelines recommend lifelong monitoring and follow-up of Phe levels and treatment adherence through home blood sampling and outpatient hospital visits [6]. This is particularly emphasised in pregnant women with PKU due to the risk of teratogenic effects associated with elevated Phe levels, such as growth retardation, microcephaly and birth defects [6,28]. Follow-up remains essential and aims to support patients and prevent long-term complications of the condition [6]. Thus, attendance at follow-up may be associated with improved metabolic control [44], with the contrary posing a possible risk of elevated mean Phe levels [15].

## 4. Protein Substitutes and Formula Composition

Phe-free L-amino acid (AA)-based substitutes are an integral part of dietary management in PKU, providing the majority of protein intake, particularly in classical PKU [34,45]. AA-based substitutes act as a primary source of substrates needed for tissue growth, repair and other important metabolic processes [46] and can be in the form of liquids or powders, which can be mixed to form a semi-solid paste [40]. Adequate consumption of such substitutes may be of particular importance in those with increased energy demand, for example, those with regular engagement in physical exercise [47]. Given that endogenous creatine synthesis can be reduced in PKU, those with PKU may also consider supplements such as creatine to support post-exercise recovery and muscle energy metabolism [47].

Prescribed amounts of AA-substitutes vary across Europe; for example, infants may be prescribed around 2–3 g/kg/day, compared to those 1–10 years being prescribed around 1.5–2 g/kg/day. This may decrease to 1 g/kg/day for children over the age of 10 years [6].

Over the years, advances to AA-based substitutes have been made to optimise both nutrition and taste, with many modern formulas containing all essential amino acids, apart from phenylalanine, vitamins and minerals [46]. In contrast, original formulas often contained insufficient nutrients, such as long-chain polyunsaturated fatty acids [48]. Adequate intake of protein substitutes is essential for maintaining Phe levels within range, with poor consumption being linked to poor metabolic control [16]. Furthermore, a diet already low in protein may result in nutritional deficiencies if not adequately supplemented with a protein substitute [16].

Compared to natural protein, AA-based substitutes are rapidly absorbed and lead to lower nitrogen retention [6]. It is due to these utilisation differences that European PKU guidelines recommend a 40% increase in the prescribed intake of AA-based substitutes [6]. In a study by Huemer et al. [49], it was found that increasing recommended daily protein intake in children aged 2 months to 15 years by a mean value of 24% did not significantly impact height, weight or BMI in comparison to controls. This study supports the hypothesis that protein quantity is not the only factor which effects growth outcomes in children with PKU, but protein quality and timing of intake [28,49]. For example, an even administration of Phe may promote improved protein utilisation, leading to better absorption and enhanced muscle protein building [45].

As well as AA-based substitutes, caseinglycomacropeptide (CGMP)-based substitutes can be used as an alternative [6,18]. GMP is a peptide originating from whey protein [50] and is naturally low in Phe [45]. When supplemented with AAs, CGMP can form the base of a protein substitute, which is used to provide a palatable alternative to AA-based substitutes as a primary protein source [51]. It has been shown that CGMP-based substitutes may provide more scope for dietary protein to be provided from intact sources such as GMP, fruits and vegetables, with synthetic AAs making up a smaller portion [16]. For example, in the traditional low-Phe diet using AA-based substitutes, these supplements provide roughly 80% of protein from synthetic sources, compared to around 30% in diets incorporating substitutes based on CGMP [16]. Furthermore, CGMP-based substitutes may decrease the absorption rate of AAs [18] and improve protein retention in comparison to AA-based substitutes [52].

Despite these potential advantages, CGMP must be supplemented with AAs to become a complete protein source and is not completely Phe-free [16]. Although a study by Ney et al. [52] did not find that CGMP-based substitutes increase plasma Phe levels in adults and adolescents, the Phe content of CGMP-formulas may negatively impact blood Phe levels and metabolic control in children [16,45]. Thus, careful consideration should be applied when prescribing to children under 12 years [45]. In addition, sufficient energy intake in those under 4 years of age should also be ensured, with the calorie content of CGMP-based substitutes varying depending on the brand [16]. These potential limitations, alongside the lack of strong evidence on CGMP formulas, highlight gaps in current knowledge and practice. As such, European PKU guidelines do not provide practical recommendations on the use of CGMP [6,28]. Some evidence suggests CGMP supplementation may have beneficial effects on appetite regulation and satiety signalling [52,53], alongside improved taste and nitrogen retention [28]. Other literature reports no significant difference in satiety and acceptability in comparison to AA-formulas [54,55]. Overall, although CGMP-based formulas show promise in potentially allowing more dietary flexibility, further research is needed in paediatric populations to evaluate their overall efficacy and use.

## 5. Natural Protein and Low Protein Specialty Foods

Though the majority of protein intake comes from AA-based substitutes in the dietary management of PKU, natural protein intake in the form of “free foods” and low-protein specialty foods are also included as part of the diet. Although parameters of protein intake are recommended, it is not uncommon that patients exceed recommended levels [20,56]. Studies by MacDonald et al. in 1996 [20] and Pinto et al. [56] demonstrate consistent findings that increasing natural protein intake in children did not negatively affect metabolic control long-term. Given the difficulty in adherence associated with the PKU diet, this may suggest that strict emphasis on exact protein intake may not be beneficial to maintaining Phe levels within range.

Special low protein foods (SLPFs) are formulated to be low in Phe and provide >30% of energy intake in children aged 5–16 years [40,57]. Examples include bread, pasta, biscuits and egg replacers [28]. SLPFs contain around 43–100% less protein than normal comparisons; however, the nutritional composition of these foods is highly variable [58]. Although the exact micronutrient profile of SLPFs is unclear, some evidence suggests higher levels of lipid and carbohydrates [59] and lower fibre content in comparison to normal food products [40]. As adherence to diet can be challenging in PKU, SLPFs are encouraged to improve variety, satiety [28], taste and acceptability [59]. Increasing the range of these foods may improve dietary adherence, especially in the teenage population [57,60].

Whilst maintaining adequate protein intake is essential for growth [6], access to various protein sources may be a barrier for those with PKU. As access and cost of SLPFs vary geographically [28,59,61,62], this may influence adherence and provide challenges in maintaining blood Phe levels [57,61]. In the UK, SLPFs are part of the national prescribing system and thus prescribed to children with PKU who are aged under 16 years, free of charge [61]. Though this provides greater access for those of a lower socioeconomic background, challenges remain. For example, Wood et al. [61] found that SLPFs were under-prescribed, with some patients reporting prescriptions being refused or containing items deemed ‘too luxurious’. Whilst increased availability and reasonable cost are favourable, careful monitoring is important as the nutritional composition of SLPFs differs nutritionally from their standard counterparts [57].

## 6. Micronutrient Requirements

As there are no PKU-specific micronutrient reference values, optimal intake of micronutrients within dietary management is not precisely defined [63]. Nutritional requirements should theoretically be met by intake of protein substitutes, as these are supplemented with the correct long-chain fatty acids, vitamins and minerals to meet dietary needs [28]. However, although children with PKU may have sufficient intake of micronutrients, they often do not achieve optimal micronutrient status [64]. Dietary non-adherence may contribute to deficiencies in micronutrients such as calcium and phosphate, a factor which may increase bone age and lower bone mineral density in children and adolescents [11,65]. Furthermore, metabolic determinants impacting absorption/excretion and the nutrient bioavailability in Phe-free formulas may also play a role in deficiencies [66]. Though deficiencies in Vitamin D, selenium, zinc, calcium or vitamin B12 may be present in a small population of children and adolescents with PKU [11,66], most present with generally adequate micronutrient status when there is good dietary adherence and regular clinical monitoring [11,66,67].

The presence of small sample sizes and variation in formula types and treatment protocols diminishes the quality of data that exists on dietary intake in PKU [66]. Even if deficiencies are identified, it is challenging to decipher if this is attributed to diet, effects of disease or compliance [11].

## 7. Dietary Adherence

It is well established that treatment adherence is challenging in PKU [6,16], often negatively impacting metabolic control. Though there is no specific measurement for compliance [15,68], there are signs that may suggest someone with PKU may be struggling to adhere to treatment during childhood. For example, poor engagement with clinicians, irregular submission of blood Phe samples and suboptimal Phe control [6]. Potential risk factors for non-adherence include parental denial or misunderstanding of PKU, lower intake of Phe-free formulas and elevated plasma Phe concentrations [15]. Though it is generally accepted that metabolic control may decrease with age [15,44,69], data regarding the relationship between age and adherence show some variation. Some studies provide evidence that Phe control may improve in adolescence [44,60,68], later deteriorating with the transition into adulthood. In a multicentre study by Alex Pinto and colleagues, it was found that Phe control was better in children below 6 years of age compared to those between 6 and 12 years [44]. In teenage participants aged 13–18 years, Phe levels increased again. This finding is consistent with a study by Ahring et al., which found that Phe levels improved during adolescence [60], before declining after the age of 16 years.

Empowerment of those caring for children with PKU is fundamental [69,70] as caregivers play a significant role in the monitoring of PKU treatment [6]. Some evidence suggests that stricter management, such as meticulous Phe measurement of all foods, may not significantly impact blood Phe control [6], highlighting the potential for flexibility in clinical management approaches. This knowledge may reduce the burden placed on children with PKU and caregivers, in turn harnessing a stronger motivation and attitude towards dietary restriction.

European PKU guidelines acknowledge that attitudes and behaviours, as opposed to knowledge alone, may improve adherence [6]. This is supported by Zamani et al. (2021), where the use of behavioural interventions targeting attitudes, motivation and social support was successful in reducing blood Phe levels in the paediatric population [70]. Methods in which this can be achieved include behavioural interventions for parents of children with PKU that focus on social support, dietary management skills, e.g., reading of food labels and self-monitoring [70]. Thus, continued research into age-specific behavioural interventions for children may be beneficial in improving metabolic control [70]. Measuring adherence accurately and consistently remains a challenge [15,68]. This difficulty in quantification may hinder the strength of conclusions and links between studies.

## 8. Adjunct and Emerging Therapies

### 8.1. Sapropterin

Sapropterin dihydrochloride, a synthetic form of the tetrahydrobiopterin (BH4) cofactor, is the first approved pharmacological therapy for the management of PKU [71]. The treatment was approved for individuals aged 4 years and over in the United States by the U.S Food and Drug Administration in 2007 [72]. After demonstrating sustained efficacy and tolerability in children under 4 years, sapropterin has more recently been expanded for use in this age group in Europe [6,22,73].

BH4 is an essential co-substrate in the conversion of Phe to tyrosine by the PAH enzyme [71] (see Figure 1). Thus, treatment with BH4 enables optimisation of enzymatic activity. Individuals with preserved PAH activity gain the greatest clinical benefit from such treatment, with responsiveness most common in mild PKU but occasionally observed in classical PKU [6,74].

To determine responsiveness to BH4 treatment, it is recommended that patients undergo a BH4 loading test before treatment initiation [6]. The loading test involves administration of a 20 mg/kg/day dose of BH4 for a minimum duration of 48 h [75]. According to European PKU guidelines, BH4 responsiveness is defined as a >30% reduction in blood Phe levels or possession of two BH4-responsive gene variants. As sapropterin facilitates lowering of Phe levels, it allows for less restriction of Phe within the diet [22,71,74,76] and a reduction in protein intake from synthetic substitutes [76]. This is beneficial to both paediatric patients and caregivers, given the burden of dietary adherence [75].

Due to limited data on the safety of BH4 use in pregnancy, the European Summary of Product Characteristics advises caution when considering use during pregnancy [77]. European PKU guidelines suggest that BH4 treatment should only be considered in PKU patients who are known to respond to BH4 and where dietary control is insufficient [6]. American guidelines also permit the use of BH4 for those who are responsive as an adjunct to dietary management in women who are pregnant or breastfeeding [8,78]. However, the presence of limited data on its use in such circumstances is acknowledged.

Pharmacological management options such as sapropterin provide an opportunity to increase dietary flexibility and thus improve quality of life in children with PKU. In line with this, those with dietary treatment supplemented with sapropterin have shown significantly decreased Phe levels compared to those with dietary treatment alone [44]. Longitudinal data have demonstrated preserved intellectual functioning, stable growth and adequate control of Phe levels in BH-4 responsive children who began treatment between the ages of 0–6 years [79]. However, many children with non-BH4-responsive PKU are unable to benefit from its advantages, highlighting a limitation of this option. This is significant as it is often children with more severe types of PKU who are most vulnerable to the condition’s neurodevelopmental and metabolic consequences, such as slower growth rates [80,81,82]. More recently, sepiapterin, a natural precursor to BH4, has been approved as a pharmacological management option for PKU [83]. This novel treatment has been shown to achieve a reduction in plasma Phe levels in those who possess both BH4-responsive and non-BH4-responsive gene variants [84], suggesting broader efficacy across genotypes.

Overall, most patients using sapropterin will still require dietary restrictions and regular monitoring of Phe levels; thus, it is not a complete solution to many of the barriers in the treatment of PKU in children.

### 8.2. Pegvaliase

Pegvaliase is an enzyme substitution therapy for PKU and is administered via subcutaneous self-injection. The medication consists of a phenylalanine ammonia lyase (PAL) enzyme conjugated with polyethylene glycol, which converts Phe to ammonia and trans-cinnamic acid [85]. Initially, a weekly dose of 2.5 mg should be administered, with the maintenance regimen consisting of 20–60 mg injections daily [85]. This therapeutic strategy may offer the potential for those with PKU to discontinue restrictive dietary practices [86,87] whilst maintaining blood Phe levels within range.

In 2018, pegvaliase was approved in America for use in adults aged 18 years and over by the U.S. Food and Drug Administration (FDA). Later in 2019, this therapy was authorised by the European Medicines Agency (EMA) for individuals aged above 16 years in Europe [85]. It is indicated in individuals with Phe concentrations > 600 micromol/L and is supported with monitoring of dietary protein, phenylalanine intake and blood Phe concentrations [85,88].

Phase 3 trials evaluating the efficacy of pegvaliase have demonstrated significantly lowered Phe levels in adult participants [88]. Furthermore, long-term follow up in an open-label extension study demonstrated sustained reduction in Phe levels and an adequate safety profile [86]. The ongoing phase 3 PEGASUS trial beginning in June 2022 has enrolled 55 participants aged 12–17 years to evaluate Pegvaliase treatment vs. dietary treatment alone [89]. According to sponsor-reported preliminary data announced in September 2025, a statistically significant decrease in blood Phe levels was observed in participants [90]. The extension phase of the PEGASUS trial is ongoing, and peer-reviewed publication of full trial data is awaited.

Although findings from the PEGASUS trial suggest potential benefit of pegvaliase for those aged 12–17 years, this management option is not yet approved for those under 16 years [91]. It is also only licenced for those with uncontrolled Phe levels > 600 micromol/L on current management [85]. With current evidence being restricted to adults, there is insufficient knowledge regarding safety and effectiveness in younger populations.

When effective, pegvaliase remains an invasive therapy and is associated with potential adverse effects such as anaphylaxis and hypersensitivity reactions [92,93].

### 8.3. Genetic Approaches

Genetic approaches including messenger ribonucleic acid (mRNA) therapy, gene addition and gene editing have been investigated in pre-clinical research as a promising treatment strategy in PKU. These methods aim to restore PAH function or integrate a PAH expression cassette into the genome [91,94].

In PKU research, gene addition approaches have utilised vectors to administer a functional PAH gene into the liver of PAH-deficient mice [95], with the most clinically effective vectors currently remaining recombinant adeno-associated virus (rAAV) vectors [91,94,96]. This method has demonstrated successful reduction in Phe levels in murine models [96,97,98]. More recently, two clinical trials by the biotechnology companies Biomarin and Homology Medicines have evaluated rAAV gene addition in adults with PAH deficiency [94]. Despite beginning enrolment, the Homology Medicines trial has since been discontinued [99]. A phase I/II clinical trial by Biomarin is currently active and has recruited adults aged 15 years and over [100]. However, data regarding trial results are not yet available.

Limitations of gene addition methods include the requirement of high vector doses and inadequate long-term efficacy due hepatocyte turnover [94]. This turnover may precipitate depletion of vector genomes with anti-AAV immunity, potentially limiting re-administration [94]. This is a particular concern in children who have increased cell turnover rates, thus long-term stability of expression is currently uncertain [94]. Alongside this, potential drawbacks also include high manufacturing costs and a theoretical risk of hepatocellular carcinoma (HCC) [94,101].

In contrast, mRNA therapy involves the administration of wild-type PAH mRNA into the bloodstream which is then translated into PAH in hepatocytes [102]. This method of gene therapy has been shown to restore PAH activity in Pah^enu2^ mice [102,103]. Although this method has been observed to successfully reduce Phe levels, this required repeat doses every 3–5 days.

A potential advantage of mRNA therapy is the direct delivery of mRNA into the hepatocyte cytoplasm, allowing for immediate translation and mitigating the risk of potentially harmful genomic integration [103]. This risk can be associated with DNA-based gene therapy, viral AAV vectors and genome editing techniques [103]. However, limitations of mRNA therapy include the short half-life of mRNA, potential immune activation, and possible entrapment of mRNA inside endosomes [94]. Although success in pre-clinical trials demonstrates proof of principle, optimisation of mRNA stability through mRNA technology may be required to develop this approach and ensure viability in humans. As of recently, there are no actively ongoing clinical trials evaluating the use of mRNA-based gene therapy for PKU. Though only in pre-clinical stages, this demonstrates promising potential for clinical application [94].

Genome-editing strategies have emerged as a possible solution in restoring hepatic phenylalanine hydroxylase activity. In contrast to gene addition approaches, gene editing alters the DNA itself and may offer a more permanent option for PKU treatment, specifically in paediatric populations [91,94]. By principle, gene editing maintains PAH expression despite genomic replication. Two methods include gene correction and gene insertion.

Gene correction methods involve the direct repair of pathogenic PAH variants, using approaches such as homology-directed repair (HDR), prime editing and base editing using CRISPR/Cas9 technology [104,105]. HDR approaches aim to correct the PAH gene mutation via inducing a double-strand DNA break nearby [105] which is then repaired via homology-directed repair mechanisms. However, evidence of this approach in PKU murine models (Pah^enu2^ mice) has shown failure to maintain a clinically effective number of gene-editing hepatocytes [105]. Thus, despite evidence showing potential in long-term therapeutic effect, PAH function has still only been partially restored, and therapeutic effects were highly variable [105].

Base editors are molecular tools that enable targeted single-nucleotide changes in DNA without creation of double-strand DNA breaks [106]. Some studies have shown base editing strategies to be effective in restoring blood Phe levels in mice [106,107]. As opposed to HDR approaches, base editing and prime editing techniques may be of higher specificity and facilitate single-base modifications in non-dividing cells [94]. Gene correction proposes strengths such the preservation of PAH regulatory control and its efficacy on dominant-negative PAH mutations [94]. However, disadvantages include the specificity of these approaches for a particular mutation, thus being a potential barrier to scalability due to the mutational heterogeneity of PKU [94].

Gene insertion is characterised by the integration of a functional PAH expression cassette into the hepatocyte genome, typically containing a PAH complementary DNA (cDNA) sequence [94]. This method has demonstrated pre-clinical success in murine models [94,108]. Using an rAAV-mediated homologous recombination strategy, Chen et al. (2020) demonstrated a ~6% insertion of human PAH cDNA into PAH alleles in humanised liver murine models [108]. Gene insertion methods may have the potential of broader applicability in comparison to gene correction, as they involve permanent insertion of a functional DNA sequence regardless of the pathogenic gene variant [94].

## 9. Conclusions

Despite recent advancements in the management of PKU in paediatric populations, lifelong dietary management remains the cornerstone of treatment. Although early dietary management is associated with improved outcomes for individuals with PKU, treatment adherence remains a central challenge. This difficulty is widely reported in the PKU literature and is particularly highlighted in later childhood and adolescence.

While pharmacological therapies such as pegvaliase and sapropterin offer the opportunity for dietary flexibility, they are not without their limitations. For example, sapropterin may only be effective in those who are BH4-responsive and pegvaliase is not yet approved for children under 16 years. Thus, a pharmacological treatment strategy which is both universally applicable and able to replace dietary practices is not yet available in the paediatric population.

Somatic gene therapy offers the potential of a more permanent treatment option for PKU, with pre-clinical data demonstrating restoration of PAH function in murine models. However, barriers such as inappropriate immune activation and high rates of hepatocyte turnover diminish clinical efficacy.

Whilst management strategies for PKU have undergone significant development, current treatment in children remains dependent on strict dietary practices which can be burdensome for both the individuals and their caregivers. Overall, further research into PAH genetic approaches and the continued development of pharmacological therapies in younger populations may expand options for long-term treatment, thus improving the quality of life for individuals with PKU.

## Data Availability

No new data were created or analyzed in this study.

## Abbreviations

The following abbreviations are used in this manuscript:

AA-based substitute	L-amino acid-based substitute
BH4	Tetrahydropterin
cDNA	Complementary DNA
CGMP-based substitute	Caseinglycomacropeptide-based substitute
EMA	European Medicines Agency
FDA	Food and Drug Administration
HCC	Hepatocellular carcinoma
HDR	Homology directed repair
HPA	Hyperphenylalaninemia
LNAA	Large neutral amino acid
mRNA	Messenger ribonucleic acid
PAH	Phenylalanine hydroxylase
PAL	Phenylalanine ammonia lyase
Phe	Phenylalanine
PKU	Phenylketonuria
qBH2	Quinonoid dihydrobiopterin
rAAV	Recombinant adeno-associated virus
SLPF	Special low protein food
